# Increasing the efficiency of digitization workflows for herbarium specimens

**DOI:** 10.3897/zookeys.209.3125

**Published:** 2012-07-20

**Authors:** Melissa Tulig, Nicole Tarnowsky, Michael Bevans, Anthony Kirchgessner, Barbara M. Thiers

**Affiliations:** 1 William and Lynda Steere Herbarium, The New York Botanical Garden, Bronx, New York, USA

**Keywords:** Herbarium specimen digitization, workflows, georeferencing, digital imaging, field books

## Abstract

The New York Botanical Garden Herbarium has been databasing and imaging its estimated 7.3 million plant specimens for the past 17 years. Due to the size of the collection, we have been selectively digitizing fundable subsets of specimens, making successive passes through the herbarium with each new grant. With this strategy, the average rate for databasing complete records has been 10 specimens per hour. With 1.3 million specimens databased, this effort has taken about 130,000 hours of staff time. At this rate, to complete the herbarium and digitize the remaining 6 million specimens, another 600,000 hours would be needed. Given the current biodiversity and economic crises, there is neither the time nor money to complete the collection at this rate.

Through a combination of grants over the last few years, The New York Botanical Garden has been testing new protocols and tactics for increasing the rate of digitization through combinations of data collaboration, field book digitization, partial data entry and imaging, and optical character recognition (OCR) of specimen images. With the launch of the National Science Foundation’s new Advancing Digitization of Biological Collections program, we hope to move forward with larger, more efficient digitization projects, capturing data from larger portions of the herbarium at a fraction of the cost and time.

## Introduction

The specimens in the world’s museums and herbaria contain a wealth of primary occurrence data that is used as the basis of many biodiversity research studies (Chapman 2005; Baird 2010; Pyke and Ehrlich 2010). Historically, herbarium specimens have only been available to researchers by visiting collections or requesting specimens on loan. Over the past 20 years, efforts have been made to make specimen data available online through the development of specimen databasing and imaging projects. While millions of specimen records are now available through institutional portals and distributed networks such as GBIF, these only represent a small fraction of the estimated 90 million herbarium specimens in the United States alone that still need to be digitized (Rabeler and Macklin 2006).

The New York Botanical Garden Herbarium (NYBG) has been digitizing its collection of an estimated 7.3 million herbarium specimens since 1995. In the first fifteen years of digitization projects, we databased 1.3 million specimens at a rate of 10 specimens an hour, leaving 6 million specimens to database. Continuing at this rate, complete digitization of the herbarium would take another 600,000 hours. Like many institutions, past digitization projects at NYBG have focused on manageable and fundable subsets of the collection ranging from 75,000–100,000 specimens that could be completed within two to three years (Vollmar et al. 2010). For example, our collection of specimens from Brazil, estimated at half a million specimens, was broken into three National Science Foundation proposals and funded over 11 years. As a result, three separate passes were made through the herbarium to locate specimens from each region of Brazil. This was an inefficient but necessary way to find the relevant specimens and complete full specimen label data entry.

With more community support for digitization of natural history collections and new programs such as the National Science Foundation’s Advancing Digitization of Biological Collections (ADBC), it is necessary to develop digitization protocols and workflows that maximize the rate of specimen digitization without sacrificing the most useful information on each specimen (Granzow-de la Cerda and Beach 2010; Scoble and Bourgoin 2010). Over the course of subsequent projects, NYBG has tried several methods to develop more efficient approaches to digitization, while still providing a high level of data quality to the scientific community who use these specimens.

## Digitization workflows

### Strategy 1: Manual data entry

Each project started with the curation of the taxa involved to reflect currently accepted names, based on recent monographs where available such as *Flora Neotropica*, on determinations by our curators and researchers visiting the herbarium, and on data available in online resources such as TROPICOS (http://tropicos.org/ ) and the International Plant Names Index (http://ipni.org/ ). During the curation phase, specimens related to the project were separated from all others with which they were filed. They were subsequently removed from the herbarium and brought to a cataloguer’s desk for data entry.

Barcodes were applied to the specimens and data entry was keyed manually from the specimen labels. Every piece of information on the label was entered, including the complete determination history of each specimen with determiners and dates. Collection information included collector, collection team, collector number and collection date. Site information included country, province or state, and county or municipio parsed separately, as well as the precise locality in a searchable text field, and geocoordinates when on the label. Habitat and plant descriptions were included word for word in text fields. Any additional notes on the label or on the sheet in general, or notes the cataloguer needed to add about the specimen, were put in other various notes fields. Authority files were also used for all taxa, and parties involved (collector, determiner, author), as well as drop down menus and look up lists for geography. Efficiencies used during this time focused primarily on organizing the specimens by collector before starting data entry to easily copy data from one record to the next. Simple measures such as encouraging cataloguers to use key strokes rather than the mouse and organizing the windows on their screen efficiently also improved data entry rates.

Staffing for these projects consisted of information managers to oversee data entry and imaging equipment, and curatorial assistants who databased and imaged the specimens. Information managers have a background in botany or biology, preferably with an emphasis in taxonomy, and several years of experience in data entry and database management. Curatorial assistants are typically new graduates in botany or biology with some herbarium experience but usually little data entry experience.

The data entry rate in this strategy averaged 10 records per hour. This rate is meant to represent an average for employing Strategy 1. It includes data entry rates from all of our major NSF projects that used this digitization approach, spanning all groups in the herbarium, and including rates of all curatorial assistants that catalogued on these projects. Only representative specimens were imaged, typically one or two per taxon.

### Strategy 2: Streamlined collection events

For the third and last leg of our Brazilian NSF projects, Species of Amazonian Brazil, we were able to leverage field book data giving us an advantage over earlier databasing projects. In the late 1970’s through the 1980’s, the New York Botanical Garden was involved in a massive collection program of the Amazonian region of Brazil, called Projeta Flora Amazonica. We retained the original field books from most of the major collectors on this project, representing roughly 80% of the herbarium’s total Brazilian Amazon holdings.

Botanists record collection data in their field books in large blocks of specimens, collected in the same site, on the same date. Often the only data different for each collection number is the taxon and plant description. Capitalizing on this, we were able to use a template tool in our database to mass enter the majority of the collection data from each field book rapidly, entering each collection event only once instead of repeatedly for each collection number as we came across each specimen in the herbarium. This also allowed us to georeference the site only once and apply it to all of the collection events.

In addition, we collaborated with the Instituto Nacional de Pesquisas da Amazônia (INPA) who had already catalogued most of their specimens. Because many of our specimens are duplicated there, we imported a subset of collection events from their database, adding to the pre-load of data compiled from the field books. This added data for an additional 10% of NYBG holdings for Amazonian Brazil.

Data entry then proceeded as with previous projects. The specimens were curated, separated and removed from the herbarium for data entry from the specimen labels. With this pre-load of data from the field books and imports, the only information to add was the taxon and plant description, and the completion of fully catalogued records increased to 30 records per hour. At this stage the records were made available online.

### Strategy 3: Semi-automated approach

With funding from the National Science Foundation’s ADBC program, our digitization strategy shifted from entering complete specimen records to entering partial records with an image for every specimen. From this point, work will be done to complete these records by several means, focusing more on automated tools to extract data from the images and by entering data from the images rather than the specimens themselves. To keep up with this new demand for images, we also upgraded our imaging protocol, as outlined below.

As with previous projects we first curate the taxa involved. This continues to be a time consuming but necessary step of the process, ensuring that the data online and in the herbarium are current. Because ADBC grants fund larger digitization projects, the usual next step of separating out project specimens has been eliminated, as we are now digitizing complete sections of the herbarium at once. This enables us to pull entire folders from the herbarium without having to separate specimens within the folders, inspect each label and make the determination as to whether or not the specimen should be included.

Using a template tool in the database, we are able to rapidly mass create partial records by barcode number range. We auto-generate the number of records based on the number of specimens we have per taxon, at a rate of 125 records per hour. This barcoding process is done in the herbarium on a cart adjacent to the cabinet in which the specimens are housed. Once they are barcoded, they are tagged and returned to the cabinets until digitization staff sweep through the cabinets and image all the specimens. While this requires us to remove specimens from the cabinets twice, we use highly-trained curatorial assistants to curate the specimens and make decisions on the current nomenclature and part-time staff or interns to image the specimens. Each staff member can then work independently, but working in teams is another approach we plan to consider.

During image processing, all images are run through optical character recognition software (OCR) to produce a text output of the specimen label. The unparsed data is then added to a fully-searchable text field in the specimen record. This will provide an initial way to search the records online until the records are completed and a mechanism for grouping records by collector or location during data entry. While we previously pre-sorted the physical specimens by collector before data entry, we will now attempt to pre-sort them via the OCR text. While not all labels contain typeface that will OCR, many that are partially handwritten have at least some typed “master label” information including collector name and some locality information, since a large portion of our herbarium was collected in the 20^th^ century.

For projects where we have a large collection of field books, such as our NSF-funded Caribbean Project, we will continue to pre-load collection events records into the database. For all other records, we will parse the OCR text using automated tools in development such as Salix, the semi-automatic label information extraction system being developed at Arizonia State University (http://nhc.asu.edu/vpherbarium/canotia/SALIX3.pdf ) and Apiary (http://www.apiaryproject.org/high-throughput-workflow-computer-assisted-human-parsing-biological-specimen-label-data ). We will also use duplicate matching applications such as FilteredPush (Wang et al. 2009) and Specify’s Scatter, Gather Reconcile (http://specifysoftware.org/content/specify-64 ). The end result will be records with the most pertinent data fully searchable in the database, including collector, collection number, date, current taxonomic name, and complete locality information. Since we are now taking an image of every specimen, any secondary data will still be available in the image of the label, or in the OCR text of the label that will still be available in a notes field. This includes plant description, habitat, and other notes found on the specimen.

**Figure 1.**
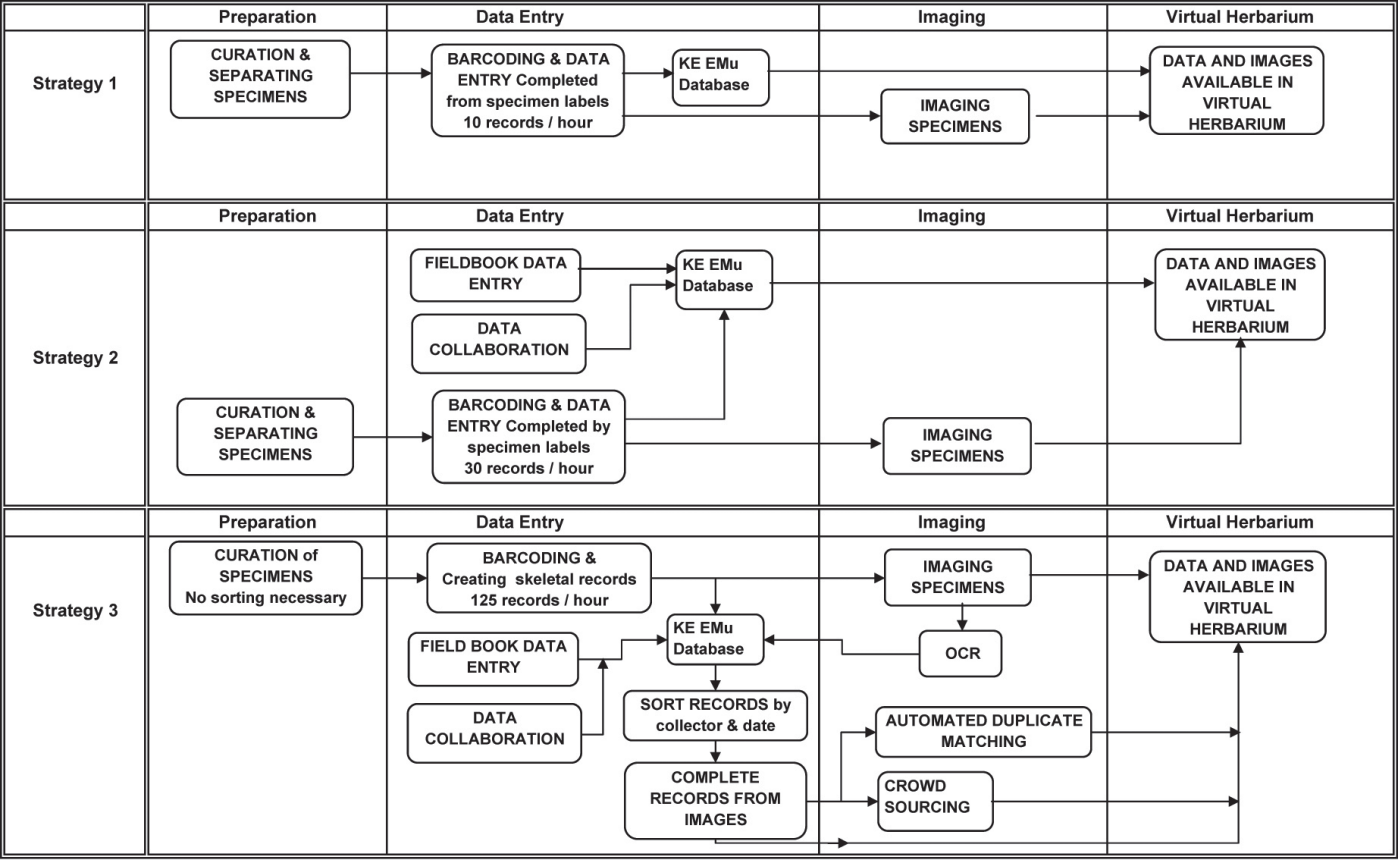
Digitization workflows at The New York Botanical Garden over the past 17 years.

## Specimen imaging

### Imaging equipment

**Figure 2.**
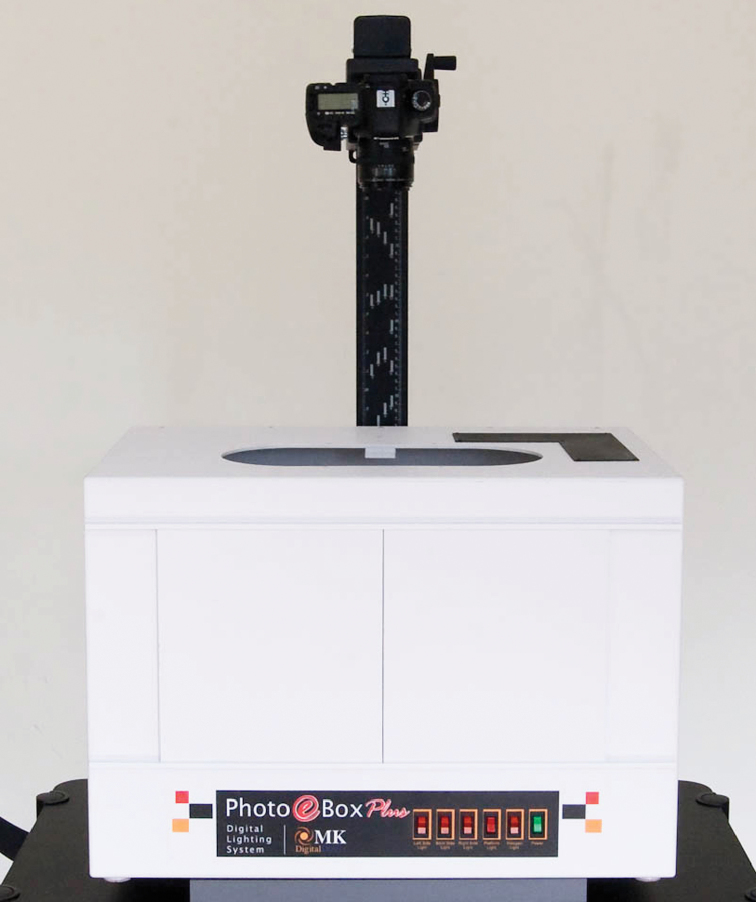
NYBG imaging station consisting of a Canon Eos 5D Mark II digital camera body, a Canon EF 50mm f/2.5 Macro lens, Photo e-Box Plus 1419 from MK Direct, and Kaiser RS 1 copystand.

To accommodate the image production expectations of the rapid digitization grants, several low cost imaging stations built with commercially available digital photography components were assembled. These components include: the Canon Eos 5D Mark II digital camera body, a Canon EF 50mm f/2.5 Macro lens, the Photo e-Box Plus 1419 from MK Direct, a Kaiser RS 1 copystand, and a Wasp bar code reader, and a laptop computer (Figure 2).

The Canon Eos 5d Mark II camera was selected to meet the image size requirements of 21 megapixels. With a resolution of 5616 × 3744 pixels, or 21.1 megapixels, the images can be enlarged on screen up to 78 × 52”, which is roughly four times the size of the original specimen sheet. The lens used is a macro lens with a normal focal length that produces little or no edge distortion. This is optimal not only for scientific study but may also produce better results when read by optical character recognition (OCR) software.

The Kaiser copystand supports the camera so that the focal plane is 31” above the specimen. This provides for a full frame image with a quarter inch border on three sides and a one inch border on the top of the specimen. A metric scale and a Munsell color target are placed in the one inch border along the top edge of the specimen.

Specimens are illuminated by placing them inside the MK Direct Photo e-Box 1419 lightbox. 5000 Kelvin fluorescent lights provide even illumination across the entire surface of the specimen with minimal heat. Supplemental 5500 Kelvin LED lighting is used to accentuate the appearance of the surface texture of the specimen.

The imaging equipment used at The New York Botanical Garden Herbarium has now become standard equipment for the National Science Foundation’s Advancing Digitization of Biological Collections project, Plants, Herbivores and Parasitoids. Identical camera work stations are being used at a number of partner institutions.

### Imaging workflow

Digitizers gather the barcoded and cataloged specimens in the herbarium then transport them to the imaging station via herbarium cart. The lightbox is powered on and allowed several minutes for the lights to stabilize and the computer and camera are powered on and the camera software is started.

A specimen is placed in the lightbox. To ensure correct alignment, a template specimen sheet is affixed to the shooting surface. The digitizer aligns the specimen with the template and shuts the front panel of the lightbox. Once the specimen is placed in the lightbox the digitizer presses the shutter release button in the camera software, taking the exposure.

The camera settings are as follows: 5000 Kelvin white balance, ISO 100, 1/60^th^ of a second shutter speed at f/9.0. To streamline image quality control and post production, all imaging workstations are configured identically in order to produce consistent images. The white balance of individual cameras may be manually modified to account for subtle differences in the color temperature of the lights.

The first image recorded is opened and inspected to confirm focus, exposure and color balance. Subsequent images are inspected periodically. Once each image is recorded, the digitizers rename the image files by scanning the barcodes on the specimens with the barcode reader. Using a rubber stamp, photographed specimens are stamped with the word “Imaged” to avoid unnecessary reimaging in the future.

The current average imaging rate is 85 exposures per hour. This means that a full time, dedicated digitizer imaging for a full 150 hours per month, could produce well over 12,000 images per month. Each image file is approximately 25 megabytes for a total of over 300 gigabytes of data monthly.

### Image quality control

Digital camera images from each imaging station are recorded in a master imaging log and the files are transferred via external hard drive to a central image quality control work station. Image quality control is performed on a single workstation with a monitor calibrated using the Xrite i1 calibrator to ensure optimal viewing. Image files are viewed and modified using Adobe Lightroom.

Image thumbnails are visually scanned en masse to confirm that the image orientation is correct and to identify any obvious defects. Periodic images are magnified to 100% magnification in order to confirm focus and that the barcode on the specimen matches the file name. Roughly every twentieth image is examined.

The image files contain technical Exchangeable Image File Format (EXIF) metadata. Additional International Press Telecommunications Council (IPTC) metadata, including Creator, Image Title, and Copyright information, is added to the image files en masse using Adobe Lightroom’s Library module.

### Image processing

Once quality control is assured, the camera files are enhanced for viewing using Adobe Lightroom’s image editing adjustment tools. One image representative of one shooting session per camera workstation is selected and modified and the modifications are applied to all other images recorded in that session.

A more precise white balance is performed by sampling the white reference on the Munsell color target included in the image. The tonality is adjusted so that the color reference target values meet manufacturer’s specification ensuring proper exposure. Sharpening is applied to enhance detail. Chromatic aberration caused by the lens is removed. For complete examples with screen shots refer to Image Editing Guidelines (http://tinyurl.com/764z7wx ).

### Archive

Once processed, the proprietary Canon digital camera files are converted to Adobe’s DNG format and copied to an archive server. Each DNG file is approximately 25 megabytes.

Tape backups are automatically made of all new files on the server. Additionally, a complete tape backup of the entire archive takes place every six months and the tapes are stored off-site.

### Access

Once saved as DNG and archived, specimen images are saved as full size, 5616 × 3744 pixel jpegs using the sRGB color space. Each jpeg is approximately 8 megabytes.

The jpegs are imported into the database where the barcode file name is matched to the corresponding catalog records and the images are made publicly available online immediately.

### Optical character recognition

The New York Botanical Garden Herbarium uses ABBYY FineReader optical character recognition software to produce text files from specimen labels. An Adobe Photoshop Action (macro) is used to automatically reduce the file size of the specimen images. Each access image is cropped in half (label data is usually found on the lower half of a specimen sheet) and converted to grayscale. This reduces the file size of each specimen to less than one megabyte. The resulting grayscale jpegs are processed using ABBYY FineReader and a separate text file for each image is saved.

The temporary grayscale images and the resulting OCR text files are returned to the catalogers. Viewing the grayscale images reduces the time required to open large files, allowing the cataloger to quickly verify the OCR text which is then manually parsed into the correct database fields. In the event that the label data is not included in the cropped area, the image may be retrieved from the database and the label data can be transcribed manually. After parsing the OCR text, the grayscale images are discarded.

### OCR text to database

A Powershell script is run to extract the data from each saved text file. The script opens Microsoft Excel and inserts a new row for each file, adding the barcode (which is read from the file name) and the label text. Since the barcode number is also part of the text itself, a comparison of the file name barcode and the text barcode can be made to reveal errors in either the file naming procedure or the OCR process.

Once the data are in Excel, they can be directly imported into the database to a searchable notes field. Rows in Excel can be grouped according to common textual information, such as a collector’s name or an expedition title. This step allows other fields in the database to be filled when the label text is imported.

## Discussion

As a result of new databasing strategies, the rate of adding specimen records to the database has gone from 10 complete records per hour to 125 partial records per hour. The resulting records have limited parsed label data initially, but are all imaged, available online immediately, and indexed by scientific name. The records will then be completed over time using the specimen image instead of the specimen itself. The result will be an index of all of our holdings for large portions of the herbarium, and eventually, for all 7.3 million specimens. It is important to note that none of these rates take into account the time put in by information management staff who oversee and train curatorial and digitization staff, import and clean database and authority files, install and troubleshoot camera equipment, process and archive images, and manage server and database upgrades.

With relatively high error rates still facing OCR and automated parsing of label data, a shift to more automated approaches has the potential to reduce the quality of information we typically provide. We feel the best first approach to complete partial records is to use database templates to mass ingest repetitive data from collector’s field books for specimens deposited at NY. For some projects, we are fortunate to have the field books for the majority of the collections. This model has the potential to be useful for a wider audience in conjunction with projects like the Smithsonian’s Field Book Project (http://www.mnh.si.edu/rc/fieldbooks/ ), which is creating an online index of these resources. Next, using duplicate matching applications such as FilteredPush and Specify’s Scatter, Gather, Reconcile to search for records already fully databased by other institutions ensure that we complete the partial records with quality information.

We will then rely on automated techniques to complete the remaining partial records from the OCR text by such applications as SALIX or APIARY. It is very likely that none of these techniques will work for the completion of all labels, especially handwritten ones. Manual transcription of data will still be necessary to complete such labels. Some of this manual transcription will be done by project staff, but we also hope to enlist volunteers, especially citizen scientists with a particular interest in using these data for their own activities or research, or as a leisure activity, to help complete the records using a crowd sourcing website that we will develop for this purpose. By combining all of these approaches, we hope to rapidly catalogue the majority of the herbarium with quality information and make these records available for other institutions to download or for use in biodiversity studies.

## Conclusion

The New York Botanical Garden Herbarium’s cataloging and imaging procedures have evolved to the point that the limiting factor in digitization is no longer technology but manpower. As we work towards our goal of digitizing the approximately 6 million specimens remaining, we hope to continue to increase our rates and learn from new developments in the biodiversity informatics community. To supplement our efforts The New York Botanical Garden is enlisting volunteers and citizen scientists whenever possible. While we can look forward to even greater advances in imaging technology, optical character recognition software, improved databasing and barcoding technologies, ensuring accurate data relies on well trained staff and an institutional commitment to the future growth of digital collections.
